# Can Routine Laboratory Tests Be Suggestive in Determining Suspicions of Malignancy in the Case of Thyroid Nodules?

**DOI:** 10.3390/medicina59081488

**Published:** 2023-08-18

**Authors:** Mervat Matei, Mihaela Maria Vlad, Ioana Golu, Cristina Ștefania Dumitru, Graziano De Scisciolo, Sergiu-Ciprian Matei

**Affiliations:** 1 Department of Doctoral Studies, “Victor Babeș” University of Medicine and Pharmacy Timișoara, Eftimie Murgu Sq. no. 2, 300041 Timișoara, Romania; mervat.hassan@umft.ro; 2 Endocrinology Clinic, “Pius Brînzeu” Emergency Clinical Hospital Timișoara, Liviu Rebreanu Boulevard no. 156, 300723 Timișoara, Romania; vlad.mihaela@umft.ro (M.M.V.); igolu25@yahoo.com (I.G.); 3 Endocrinology Department, “Victor Babeș” University of Medicine and Pharmacy Timișoara, Eftimie Murgu Sq. no. 2, 300041 Timișoara, Romania; 4 Department of Microscopic Morphology/Histology, Angiogenesis Research Center, “Victor Babeș” University of Medicine and Pharmacy, Sq. Eftimie Murgu no. 2, 300041 Timișoara, Romania; 5 Faculty of Medicine, “Victor Babeș” University of Medicine and Pharmacy Timișoara, Eftimie Murgu Sq. no. 2, 300041 Timișoara, Romania; graziano.descisciolo@student.umft.ro; 6 Abdominal Surgery and Phlebology Research Center, “Victor Babes” University of Medicine and Pharmacy, Eftimie Murgu Sq. no. 2, 300041 Timișoara, Romania; matei.sergiu@umft.ro

**Keywords:** thyroid nodules, thyroid cancer, laboratory tests, usual analysis

## Abstract

*Background and objectives:* Thyroid nodules are a common finding in clinical practice and can be either benign or malignant. The aim of this study was to compare laboratory parameters between patients with malignant thyroid nodules and those with benign thyroid nodules. *Materials and methods:* A total of 845 patients were included, with 251 in the study group (malignant thyroid nodules) and 594 in the control group (benign thyroid nodules). *Results:* Our results show that there were statistically significant differences in several laboratory parameters, including FT3, FT4, ESR, fibrinogen, WBC, and lymphocyte percentage, between the two patient groups (*p* < 0.05). *Conclusions:* These findings suggest that certain laboratory parameters may be useful in differentiating between benign and malignant thyroid nodules and could aid in the diagnosis and treatment of thyroid cancer. However, further diagnostic tests such as fine-needle aspiration biopsy and imaging studies are typically required for an accurate diagnosis. Routine laboratory tests prove most effective when combined with other diagnostic methods to identify thyroid cancer. Although not conclusive on their own, these tests significantly suggest and guide physicians to suspect malignancy in thyroid nodules. This affirmative answer to our question, “Can routine laboratory tests be suggestive in determining suspicions of malignancy in the case of thyroid nodules?” aligns with the results of our study.

## 1. Introduction

Thyroid nodules are common in the general population, with a higher prevalence in women and with advancing age [1]. Their importance lies in the need to assess thyroid function, the degree of and future risk of mass effect, and exclude thyroid cancer, which occurs in variable percentages of thyroid nodules [2]. Thyroid cancer is the most common malignant endocrine tumor [3]. The increasing incidence and financial burden of thyroid cancer have drawn widespread attention. Its incidence has increased by approximately 10% annually over the past 30 years. According to Global Cancer Statistics, thyroid cancer ranks ninth among 36 cancers globally [4], being at the same time the fifth most common cancer in women in the USA [5].

Ultrasonography is the standard technique to assess the underlying thyroid parenchyma, and it is the optimal non-invasive imaging modality to determine which nodules demonstrate malignant features and evaluate for abnormal cervical lymphadenopathy [6]. Various risk stratification systems exist to categorize the risk of malignancy based on the ultrasound appearance of a thyroid nodule. Nodules are selected for fine-needle aspiration biopsy based on ultrasound features, size, and high-risk clinical history. Regarding risk stratification systems, TIRADS (Thyroid Imaging Reporting and Data System) and R-TIRADS (Revised TIRADS), which are 5-point scoring systems for thyroid nodules on ultrasound, are often used. Those scoring systems help to decide if a thyroid nodule is benign or malignant by combining multiple ultrasound features and enabling physicians to diagnose thyroid nodules more efficiently. By using this method, the number of unnecessary fine-needle aspirations can be considerably reduced [7].

Even though fine-needle aspiration biopsy is an effective method to discriminate malignant thyroid nodules, it achieves indeterminate results in approximately 30% of cases [8]. Cytology results are classified by the Bethesda system into six categories ranging from benign to malignant [9]. When cytology is indeterminate, molecular testing can improve the diagnostic accuracy of fine-needle aspiration and further stratify patients for observation or surgery [1,8]. However, these tests often have high costs. Based on the US health care system, the total cumulative costs incurred by the thyroid cancer diagnosis increased to more than $2.38 billion in 2019 [4]. Due to the costs, in countries with low and medium incomes, such expensive investigations are not performed on all patients but only in selected cases. Instead, a series of routine blood tests is usually requested for all patients with an endocrinological follow-up.

Routine laboratory tests, which are usually not expensive, are performed for almost all inpatients and outpatients [10]. Although it has been observed that an increased neutrophil-to-lymphocyte ratio is associated with malignant thyroid tumors [11], to our knowledge, no comprehensive study has examined other potential variations in the usual parameters of laboratory analyses that could be correlated with the malignant morphology of thyroid nodules. This study aims to analyze the variation in a series of routine laboratory parameters depending on the benign or malignant subtype of thyroid nodules.

The use of laboratory parameters, including complete blood counts, thyroid function tests, and inflammatory markers, has gained attention in recent years. These tests provide valuable information about the physiological status of the thyroid gland and the presence of associated systemic inflammation, which may be indicative of malignancy. Various studies have investigated the potential association between routine laboratory parameters and the likelihood of malignancy in thyroid nodules [12].

WBC (white blood cell) count, lymphocyte count and percentage, fibrinogen levels, erythrocyte sedimentation rate (ESR), and thyroid hormone levels (FT3, FT4, and TSH) have been extensively studied in relation to thyroid nodules [13]. The rationale behind examining these parameters lies in the fact that thyroid cancer can induce an immune response and lead to alterations in the hematological and inflammatory profiles. Previous research has demonstrated significant associations between certain laboratory parameters and the presence of thyroid malignancy. For instance, elevated WBC count, lymphocyte count, and percentage have been reported in patients with malignant thyroid nodules compared to those with benign nodules. Similarly, increased levels of fibrinogen and ESR have been observed in patients with thyroid cancer. Moreover, abnormal thyroid hormone levels, particularly elevated FT3 and FT4, have been linked to an increased risk of thyroid malignancy [14,15]. Although several advanced molecular tests based on multiple molecular markers are available for clinical use and have increased their impact on the clinical management of patients, they are not widely available. Among them is BRAF V600E (v-raf murine sarcoma viral oncogene homolog B1), one of the most studied mutations [16,17].

While these findings are promising, it is important to note that routine laboratory tests alone cannot provide a definitive diagnosis of thyroid cancer. In the evaluation of thyroid nodules, the cytopathology of thyroid fine-needle aspiration specimens plays a central role [18]. Fine-needle aspiration biopsy (FNAB) remains the gold standard for confirming malignancy and guiding further management decisions and should be performed in any case where the TIRADS score raises the suspicion of a malignant thyroid nodule. However, integrating laboratory parameters with other diagnostic modalities, such as imaging and FNAB, may enhance diagnostic accuracy and help in risk stratification [19].

Considering the potential utility of routine laboratory tests in evaluating thyroid nodules, this study aimed to analyze the association between various laboratory parameters and the likelihood of malignancy in a cohort of patients with thyroid nodules. By assessing these laboratory parameters alongside clinical characteristics and imaging findings, a more comprehensive approach to the diagnosis and risk stratification of thyroid nodules can be achieved.

## 2. Materials and Methods

*Patients and laboratory tests*. The present study was an observational, retrospective cohort study that monitored the variations in laboratory parameters in patients with thyroid nodules. Blood test results and clinical charts of 1032 patients that presented to the Endocrinology Clinic, ”Pius Brînzeu” Emergency Clinical Hospital Timișoara, Romania, who were diagnosed with thyroid nodules and subsequently operated in the 1st Surgical Clinic, same hospital, between January 2017 and February 2023, were evaluated (all patients who presented with thyroid nodules during the defined period were initially included). Depending on the indication, various surgical interventions were performed, as follows: adenomectomy was practiced in 6 cases, lobectomy in 188 cases, and total thyroidectomy in 651 cases, respectively. Before the surgery, venous blood samples were collected from the elbow or anterior antebrachial region veins for laboratory tests. All surgically resected specimens were sent for histopathological examination following standardized procedures. The tissue specimens were manually processed for microscopic analysis. This involved sequential steps, including dehydration in alcohol solutions, clearing, paraffin embedding, and sectioning using a semi-automatic rotary microtome (Medite M530) to obtain sections with a thickness of 4 μm. Subsequently, the tissue sections were stained using the Hematoxylin-Eosin stain. The staining process utilized Bio Optica reagents and was carried out using an automatic Leica Autostainer XL Stainer, adhering to established protocols. The resulting stained slides were examined by a team of at least two pathologists using a Zeiss Axioskop 2 Plus microscope. The histopathological assessments were made collaboratively by the pathologists, and the results represent the consensus conclusions of the team. The parameters considered were age, red blood cell (RBC) count, white blood cell (WBC) count, total number and percentage of neutrophils and lymphocytes, neutrophil-to-lymphocyte ratio (NLR)—calculated for absolute and percentage values—total number of monocytes, eosinophils, and basophils, platelet count (PLT), glycaemia, fibrinogen, erythrocyte sedimentation rate (ESR), free triiodothyronine (FT3), free thyroxine (FT4), thyroid stimulating hormone (TSH), carcinoembryonic antigen (CEA), carbohydrate antigen 19-9 (CA 19-9), cancer antigen 15-3 (CA 15-3), cancer antigen 125 (CA-125), and pathological exam of the resected specimen result (benign or malignant nodule).

Considering the paraclinical parameters studied, the laboratory reference values were as follows: RBC, 4.55.9 × 106/µL; WBC, 49.5 × 10³/µL; neutrophils, 1.8–6.7/*103/μL; lymphocytes, 0.8–3.8/*103/μL; monocytes, 0.1–0.9/*103/μL; eosinophils, 0–0.4/*103/μL; basophils, 0–0.1/*103/μL; neutrophil percentage, 4570%; lymphocyte percentage, 20–40%; PLT, 150,400 × 10³/µL; ESR, 015 mm/h; fibrinogen, 200,393 mg/dL; glycaemia, 74,106 mg/dL; FT3, 3.54–6.47/pmol/L; FT4, 11.50–22.70/pmol/L; and TSH, 0.55–4.78/mIU/L.

*Enrolment criteria*. In order to establish uniformity among and between the groups, the following subjects were excluded from the study: patients with incomplete data; patients with acute or chronic inflammatory diseases; patients diagnosed, undergoing treatment, or previously treated for various cancers; patients with values of CEA, CA 19-9, CA 15-3, or CA 125 outside the reference limits of the laboratory, regardless of the confirmation of the diagnosis of a proliferative process; and patients who did not sign the written informed consent form. Furthermore, recognizing the potential impact of nodule size on the comparability of study groups, additional considerations were made. Patients presenting with thyroid nodules exhibiting significant variations in size were deliberately excluded to eliminate the potential influence of nodule size differences as a confounding factor. In this context, the study exclusively included patients with thyroid nodules ranging in size from 1 to 2.5 cm. By applying these criteria, 845 patients were finally included in the study. Subsequently, depending on the histopathological result obtained after the analysis of the surgically resected specimens, patients were assigned to a study group (251 cases with malignant thyroid tumors) or a control group (594 cases with benign thyroid tumors). The tumor types were further subclassified based on their histological diagnoses, allowing for a more detailed analysis. The following surgery types were performed: adenomectomy was practiced in 6 cases, lobectomy in 188 cases, and total thyroidectomy in 651 cases, respectively.

*Data analysis*. Statistical analyses were completed using MedCalc^®^ Statistical Software version 20.118 (MedCalc Software Ltd., Ostend, Belgium; 2022). The results were statistically analyzed using the independent sample t-test. The Kolmogorov-Smirnov test was used to analyze the normal distribution of variables. A resulting *p*-value of <0.05 was considered statistically significant and was assessed at a 95% confidence interval.

## 3. Results

From a total of 845 patients, we obtained a control group (n = 594) and a study group (n = 251), which contained both female and male patients. Of all patients in the study group, 82.8% were female, with an age range of 17–84 years (mean 46.4). The control group had 90.9% females, with an age range of 19–82 years (mean 56.41). When laboratory parameters were compared between the two patient groups, FT4 and FT3 were significantly higher in the study group (FT4 *p* = 0.0131; FT3 *p* = 0.0018). ESR and fibrinogen showed a significant correlation between the two groups (ESR *p* = 0.002; fibrinogen *p* = 0.0238), with significantly higher values in the study group. Another laboratory parameter that showed a statistically significant correlation was the WBC count (*p* = 0.0486). The percentage of lymphocytes was significantly higher in the study group (*p* = 0.027), although the difference was not statistically significant for lymphocytes (*p* = 0.669). However, there was no significant difference in the percentage of neutrophils, monocytes, eosinophils, basophils, or other laboratory parameters (Table 1).

**Figure 1:**
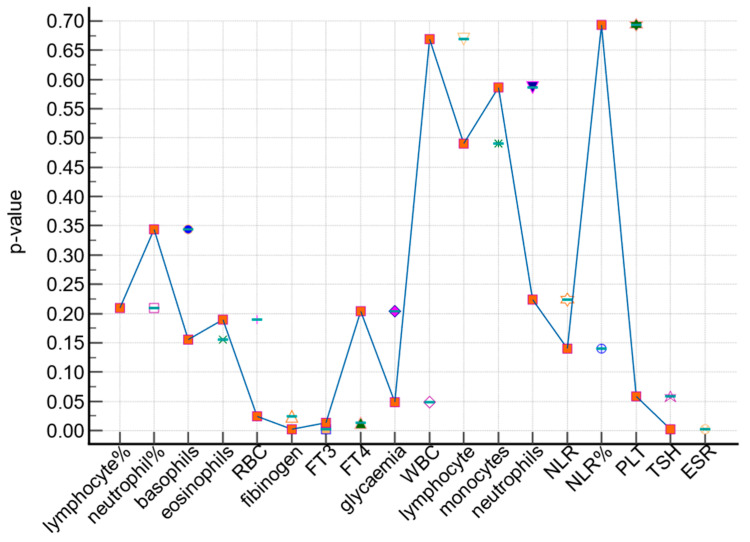
Comparison of the patients in the study group and the control group in terms of significant laboratory parameters. Different shapes correspond to each laboratory analysis according to the *p*-value, *p*-value < 0.05 = significant.

The study group (malignant thyroid nodules) and control group (benign thyroid nodules) were compared in terms of significant laboratory parameters (Figure 1). The following parameters showed statistically significant differences between the two groups at a *p*-value < 0.05: FT3, FT4, VSG, fibrinogen, WBC, and lymphocyte%. Specifically, FT3 and FT4 levels were significantly higher in the study group compared to the control group. No significant differences were observed between the two groups for other laboratory parameters.

The study group consisted of 251 patients with malignant thyroid tumors, encompassing various histological subtypes. These subtypes included Papillary Thyroid Cancer (PTC) (n = 133), Follicular Thyroid Cancer (FTC) (n = 33), Follicular Variant of Papillary Thyroid Carcinoma (n = 57), Hürthle Cell Carcinoma (n = 11), Anaplastic Thyroid Carcinoma, Medullary Thyroid Carcinoma (n = 2), Follicular Thyroid Cancer and Hürthle Cell Carcinoma (n = 3), Medullary Thyroid Carcinoma (n = 5), Papillary and Follicular Thyroid Carcinoma (n = 3), Hürthle Cell (Oxyphilic) Papillary Thyroid Carcinoma (n = 5), and Poorly Differentiated Thyroid Cancer (n = 1). Each subtype’s distinct characteristics were considered during the analysis. In the control group, composed of 594 cases of benign thyroid tumors, further differentiation was made based on the specific histopathological diagnoses. The subtypes included Nodular Goiter (n = 394), Follicular Adenoma (n = 43), Chronic Autoimmune Thyroiditis (n = 91), Graves-Basedow Disease (n = 43), Hyperfunctioning Thyroid Adenoma (n = 10), and Toxic Thyroid Adenoma (n = 13). Additionally, we analyzed the main tumor subgroups for information on how different tumor types might uniquely interact with measured laboratory parameters (Table 2).

## 4. Discussion

Thyroid nodules are a common clinical finding, with a prevalence of up to 68% in the general population, as detected by imaging studies. Although the majority of these nodules are benign, around 5–15% of cases may indicate thyroid cancer. Accurate differentiation between benign and malignant nodules is crucial for appropriate management and treatment decisions. Although imaging modalities, such as ultrasound, play a pivotal role in the initial evaluation of thyroid nodules, they have limitations in distinguishing between benign and malignant nodules. As a result, additional diagnostic tools are necessary to establish a definitive diagnosis and guide treatment strategies. Routine laboratory tests, which are easily accessible and cost-effective, have been explored as potential indicators for differentiating between benign and malignant thyroid nodules [20,21].

Patients were selected to be part of either the study group (those with malignant thyroid nodules) or the control group (those with benign thyroid nodules) based on their pathological test results. The hospital’s standard procedure involves using hematoxylin-eosin staining for all tissue samples. If the initial results give indications of a potentially malignant tumor or if there is uncertainty about the tumor’s malignancy, additional tests using immunohistochemical analyses are conducted to confirm the diagnosis [22].

In this study, our focus was on the results obtained from microscopic analysis using hematoxylin-eosin staining. This method helped us establish a clear diagnosis, especially considering factors such as the size of the tumor (ranging from 1 to 2.5 cm) and the surgical technique used to extract the whole or partially removed thyroid tissue along with the tumor. To maintain consistency in our data presentation, we decided to determine the tumor’s subtype (malignant or benign) solely based on the initial pathological findings using hematoxylin-eosin staining. It is important to note that all the pathological results we examined in our study are highly reliable. The comparison of laboratory parameters between the study group (patients with malignant thyroid nodules) and the control group (patients with benign thyroid nodules) revealed several interesting findings related to white blood cell (WBC) count, neutrophils, lymphocytes, and the neutrophil-to-lymphocyte ratio (NLR). 

The white blood cell (WBC) count is an important laboratory parameter that reflects the body’s immune response and inflammatory status. In the context of thyroid nodules, alterations in WBC count have been investigated as a potential marker for distinguishing between benign and malignant thyroid cancer [23,24].

In the present study, our findings revealed a statistically significant difference in WBC count between patients with malignant thyroid nodules and those with benign nodules. The study group, composed of individuals with malignant thyroid nodules, exhibited a higher mean WBC count compared to the control group (*p* = 0.0486). This suggests that an elevated WBC count may be associated with an increased likelihood of malignancy in thyroid nodules [25]. Several mechanisms may explain the relationship between WBC count and thyroid malignancy. Thyroid cancer can trigger an immune response, leading to inflammation and the subsequent recruitment of immune cells, including leukocytes. It is plausible that the presence of malignant thyroid nodules stimulates a systemic inflammatory response, resulting in an elevated WBC count [26].

Furthermore, we analyzed the percentage of neutrophils and lymphocytes in both groups. Although there was no significant difference in the percentage of neutrophils, the study group exhibited a higher percentage of lymphocytes compared to the control group. Lymphocytes play a crucial role in the immune response and are involved in recognizing and eliminating abnormal cells, including cancer cells. Alterations in lymphocyte count and percentage have been investigated as potential markers for distinguishing between benign and malignant thyroid cancer [27].

In our study, we observed a statistically significant difference in the percentage of lymphocytes between patients with malignant thyroid nodules and those with benign nodules (*p* = 0.027). The study group, consisting of individuals with malignant thyroid nodules, exhibited a higher mean lymphocyte percentage compared to the control group. However, there were no significant differences in the absolute lymphocyte count between the two groups (*p* = 0.669). These findings suggest that the proportion of lymphocytes within the total white blood cell population may be associated with the likelihood of thyroid malignancy, while the absolute count may not be as informative.

The higher lymphocyte percentage observed in the study group could be attributed to the immune response triggered by the presence of malignant thyroid nodules. The immune system recognizes the abnormal cells and attempts to eliminate them through increased production and recruitment of lymphocytes. This phenomenon may result in a higher percentage of lymphocytes in the peripheral blood. Further research is necessary to better understand the underlying mechanisms and clinical implications of altered lymphocyte percentages in thyroid cancer. Additionally, larger studies involving diverse patient populations are needed to validate the significance of lymphocyte percentage as a diagnostic marker for thyroid malignancy [28,29].

The neutrophil-to-lymphocyte ratio (NLR) has emerged as a valuable marker in cancer research due to its association with systemic inflammation and immune response. The underlying mechanisms that link the NLR to cancer development and progression are complex and multifaceted. In the context of thyroid cancer, the involvement of the NLR in cancer mechanisms can be attributed to several factors [30].

Firstly, chronic inflammation has been recognized as a key contributor to cancer development. Inflammatory cells, including neutrophils and lymphocytes, play a critical role in the tumor microenvironment. Neutrophils release various pro-inflammatory molecules, such as cytokines and chemokines, which can promote tumor growth, angiogenesis, and metastasis [31]. On the other hand, lymphocytes, particularly cytotoxic T cells and natural killer (NK) cells, possess anti-tumor properties by recognizing and eliminating cancer cells. Thus, an elevated NLR may reflect an imbalance between pro-inflammatory neutrophils and anti-tumor lymphocytes, favoring a pro-tumor environment [32].

Secondly, neutrophils have been implicated in the suppression of lymphocyte function. Neutrophils can release immunosuppressive factors that impair lymphocyte activation and proliferation, dampening the anti-tumor immune response. This interaction between neutrophils and lymphocytes can contribute to immune evasion by cancer cells and promote tumor growth [33].

Furthermore, the increase in the NLR observed in thyroid cancer patients may be influenced by tumor-related factors. Thyroid cancer cells can secrete chemokines and cytokines that recruit and activate neutrophils while suppressing lymphocyte activity. Additionally, cancer cells may induce changes in the bone marrow microenvironment, leading to altered production and release of neutrophils and lymphocytes, further contributing to the elevated NLR. It is important to note that the NLR is not specific to thyroid cancer and can be influenced by various factors, including infections, inflammatory diseases, and comorbidities. Therefore, it should be interpreted in conjunction with other clinical and laboratory parameters to avoid potential confounding effects [34].

In our study, the NLR showed no significant difference between the study and control groups (*p* = 0.2242). Although not statistically significant, it is worth noting that the NLR tended to be higher in the control group, indicating a potential role for this ratio in distinguishing benign from malignant thyroid nodules. Further research with larger sample sizes is warranted to explore the clinical utility of the NLR in thyroid cancer. The elevated NLR in thyroid cancer patients reflects the interplay between chronic inflammation, the immune response, and tumor-related factors. Understanding the scientific involvement of the NLR in cancer mechanisms can contribute to the development of novel diagnostic and therapeutic approaches that target the inflammatory and immune components of thyroid cancer [35].

In our study, we did not observe significant differences in red blood cell (RBC) levels between patients with benign and malignant thyroid nodules. However, it is important to consider the potential involvement of RBCs in the context of thyroid cancer. RBCs play a crucial role in oxygen transport, delivering oxygen to tissues and organs [36]. In cancer, including thyroid cancer, alterations in tissue oxygenation and blood supply can influence tumor growth and progression. The tumor microenvironment can become hypoxic due to an inadequate blood supply, leading to the activation of hypoxia-inducible factors (HIFs) and subsequent changes in gene expression. These changes may promote angiogenesis (the formation of new blood vessels) and enable tumor cells to adapt to low oxygen conditions. In thyroid cancer, studies have suggested that hypoxia-inducible factors, particularly HIF-1α, are associated with tumor aggressiveness and metastasis. HIF-1α can stimulate the production of vascular endothelial growth factor (VEGF), which promotes angiogenesis and the formation of new blood vessels to supply the growing tumor. Additionally, HIF-1α can regulate the expression of genes involved in metabolism, cell survival, and invasiveness, contributing to the overall malignant phenotype [37,38]. While our study did not find significant differences in RBC levels between benign and malignant thyroid nodules, it is possible that alterations in tissue oxygenation and blood supply may occur locally within the tumor microenvironment rather than being reflected in systemic RBC levels. Moreover, it is important to note that RBC levels alone may not be sufficient to capture the complex interplay between tumor hypoxia, angiogenesis, and thyroid cancer progression. Additional investigations, such as assessing hypoxia-related biomarkers or conducting immunohistochemical analyses on tumor tissue, may provide more insights into the involvement of RBCs in thyroid cancer.

Monocytes are immune cells involved in the innate immune response. They can infiltrate the tumor site and differentiate into tumor-associated macrophages (TAMs). TAMs play a crucial role in tumor progression and metastasis by promoting angiogenesis, suppressing anti-tumor immunity, and facilitating tissue remodeling. While our study did not find significant differences in monocyte levels, investigating the phenotypic and functional characteristics of monocytes and TAMs in thyroid cancer may provide valuable insights into the tumor microenvironment and potential therapeutic targets [39].

Eosinophils and basophils are granulocytes involved in allergic reactions and immune responses against parasites. Their involvement in thyroid cancer is not well studied, and their presence in the tumor microenvironment remains unclear. However, emerging evidence suggests that eosinophils and basophils may have a role in modulating the immune response within the tumor, potentially influencing tumor progression and response to treatment [40,41].

Platelets are critical for hemostasis and have been implicated in cancer progression, metastasis, and angiogenesis. In the tumor microenvironment, platelets can interact with cancer cells, promoting their survival, migration, and invasion. Additionally, platelets release various growth factors, such as platelet-derived growth factor (PDGF) and vascular endothelial growth factor (VEGF), which contribute to tumor angiogenesis. While PLT levels did not show significant differences in our study, exploring the role of platelets in thyroid cancer progression and their potential as prognostic markers or therapeutic targets is warranted [42].

Alterations in blood glucose levels, reflected by glycemia, have been associated with cancer development and progression. Cancer cells often exhibit increased glucose uptake and utilization to support their metabolic demands, known as the Warburg effect. Glycemia levels may reflect the metabolic state of the tumor and its interaction with the host’s systemic metabolism. Nevertheless, the significance of glycemia in thyroid cancer remains uncertain, necessitating additional research to elucidate its implications and potential contributions to diagnosis, prognosis, and treatment strategies [43]. In our study, we observed significant differences in erythrocyte sedimentation rate (ESR) and fibrinogen levels between patients with benign and malignant thyroid nodules. These findings suggest potential involvement of ESR and fibrinogen in the context of thyroid cancer.

ESR is a non-specific marker of inflammation and is influenced by various factors, including the presence of cytokines, acute-phase reactants, and changes in plasma protein composition. In cancer, including thyroid cancer, chronic inflammation is known to play a role in tumor development, progression, and metastasis. Tumor cells can release pro-inflammatory cytokines and chemokines, which stimulate the production of acute-phase reactants and activate the immune response [44].

Thyroid cancer itself can trigger an inflammatory response characterized by the infiltration of immune cells into the tumor microenvironment. This immune cell infiltration can lead to the release of cytokines such as interleukin-6 (IL-6) and tumor necrosis factor-alpha (TNF-α), which are known to influence ESR levels. Elevated ESR levels in thyroid cancer patients may reflect the presence of tumor-associated inflammation and the systemic effects of the immune response [45].

Fibrinogen, a plasma protein involved in blood coagulation, has also been implicated in cancer development and progression. Inflammation, as observed in cancer, can activate the coagulation system, leading to an increase in fibrinogen levels. Fibrinogen not only contributes to blood clotting but also promotes tumor growth, angiogenesis, and metastasis. It can interact with various cellular receptors, cytokines, and growth factors, modulating tumor cell adhesion, migration, and invasion. In thyroid cancer, elevated fibrinogen levels have been associated with tumor aggressiveness, lymph node metastasis, and a poorer prognosis. Fibrinogen can enhance tumor cell proliferation, facilitate angiogenesis, and promote the formation of a supportive microenvironment for tumor growth. Additionally, it has been suggested that fibrinogen may interact with immune cells and modulate the anti-tumor immune response, thereby influencing tumor progression [46].

The significant differences in ESR and fibrinogen levels observed in our study suggest the potential involvement of inflammation and coagulation pathways in thyroid cancer. The activation of these pathways may contribute to tumor growth, invasion, and metastasis. ESR and fibrinogen levels could serve as useful biomarkers for assessing the inflammatory and coagulation status of thyroid cancer patients, aiding in prognosis determination and treatment decision-making.

Thyroid hormones are very important in the regulation of various physiological processes, including metabolism, growth, and development [47]. Altered thyroid hormone levels have been observed in several types of cancer, including thyroid cancer. In our study, we observed higher levels of FT3 and FT4 in the study group (malignant thyroid nodules) compared to the control group (benign thyroid nodules).

Increased levels of FT3 and FT4 in thyroid cancer patients can be attributed to several factors. Firstly, thyroid cancer cells themselves can produce and secrete thyroid hormones, leading to increased circulating levels. Secondly, increased thyroid hormone levels may result from disrupted feedback mechanisms in the hypothalamic-pituitary-thyroid (HPT) axis. Tumor cells may interfere with the normal negative feedback loop that regulates thyroid hormone production, leading to uncontrolled hormone synthesis [48,49].

Thyroid-stimulating hormone (TSH), produced by the pituitary gland, regulates the release of thyroid hormones from the thyroid gland [50]. In our study, we did not observe a significant difference in TSH levels between the study and control groups. The reference range for TSH levels used in clinical practice may not adequately capture subtle alterations in hormone levels associated with thyroid cancer. The reference range is typically established based on the general population and may not account for variations specific to thyroid cancer patients. Therefore, TSH levels within the reference range may still be indicative of abnormal thyroid function in the context of thyroid cancer [51].

It is also important to consider the heterogeneity of thyroid cancer subtypes. Different subtypes of thyroid cancer, such as papillary, follicular, medullary, and anaplastic thyroid carcinoma, can exhibit variations in their impact on thyroid hormone regulation. Each subtype may have different mechanisms of action and influences on the HPT axis, which can contribute to the variability in TSH levels observed in thyroid cancer patients [52,53].

Lastly, the timing of TSH measurement in relation to disease progression and treatment interventions may influence the observed results. Thyroid cancer management often involves surgical removal of the thyroid gland, radioactive iodine treatment, or thyroid hormone replacement therapy. These interventions can affect TSH levels and complicate the interpretation of the results. The timing of TSH measurement in relation to these interventions may influence the observed TSH levels and their significance in differentiating between benign and malignant thyroid nodules [54].

Our study focused on variations in laboratory parameters in patients with thyroid nodules, distinguishing between those with benign and malignant thyroid tumors. The inclusion of a substantial cohort, composed of 845 patients, facilitated robust analyses. We observed notable differences between the study group (malignant thyroid nodules) and the control group (benign thyroid nodules), particularly in FT3, FT4, ESR, fibrinogen, WBC, and lymphocyte%, which were significantly correlated. These changes highlight the potential of laboratory parameters as indicators to distinguish between benign and malignant thyroid nodules.

Surprisingly, the analysis extended beyond broad classifications to encompass specific tumor subtypes within both groups. Within the study group, we explored a range of malignant thyroid tumor subtypes, such as papillary thyroid cancer (PTC), follicular thyroid cancer (FTC), and others. Similarly, in the control group, there were different benign tumor subtypes, such as nodular goiter, follicular adenoma, and others. We evaluated from the two study groups the most encountered tumor subtypes in our groups (Papillary Thyroid Cancer (n = 133) and Nodular Goiter (n = 394)), providing information on their interactions with laboratory parameters. The results of these two tumor types agreed with the results obtained in the study and control groups.

The influence of nodule size on the comparability of study groups is a crucial consideration in thyroid nodule research. In our study, we took deliberate steps to account for this potential confounding factor. Recognizing that variations in nodule size could introduce bias, we opted to exclude patients with thyroid nodules exhibiting substantial size differences. This strategic decision aimed to ensure that our findings accurately reflect the influence of laboratory parameters on thyroid nodule classification, rather than being skewed by the impact of nodule size. Nodule size holds paramount importance in thyroid nodule evaluation for several reasons. First and foremost, larger nodules might harbor distinct physiological and biochemical characteristics compared to smaller ones, potentially leading to variations in laboratory parameter values. For instance, a larger nodule might exhibit altered hormone secretion patterns or vascular supply, contributing to differences in measured values [55]. By deliberately restricting our study to thyroid nodules within a specific size range (1 to 2.5 cm), we sought to minimize the potential influence of nodule size on our results. This stringent inclusion criterion enhances the internal validity of our findings and supports more accurate comparisons between benign and malignant nodules. In addition to laboratory parameters, there are several other methods available for the diagnostic evaluation of thyroid nodules. These methods aim to provide additional information to complement laboratory tests and aid in the accurate diagnosis of benign or malignant thyroid nodules. Some of the commonly used diagnostic methods include imaging techniques, molecular testing, and fine-needle aspiration biopsy (FNAB) [56].

Imaging techniques such as ultrasound, computed tomography (CT), magnetic resonance imaging (MRI), and nuclear medicine scans can provide valuable information about the size, location, and characteristics of thyroid nodules. Ultrasound is widely used as an initial imaging modality for evaluating thyroid nodules due to its accessibility, cost-effectiveness, and ability to distinguish between solid and cystic nodules. CT and MRI may be employed for further characterization of nodules or to evaluate the presence of lymph node involvement or distant metastases [57].

Molecular testing, including genetic and molecular marker analysis, has emerged as a promising approach for improving the diagnostic accuracy of thyroid nodules. Techniques such as gene expression profiling, mutation analysis, and next-generation sequencing can help identify specific genetic alterations associated with malignancy. These tests can provide information about the risk of malignancy and help guide decision-making regarding the need for surgery or further surveillance [58].

Fine-needle aspiration biopsy (FNAB) remains the gold standard for evaluating thyroid nodules and determining their malignant potential. FNAB involves the use of a thin needle to collect cells or fluid from the nodule, which are then examined under a microscope by a pathologist. The analysis of FNAB samples can provide crucial information regarding the cellular characteristics, presence of malignancy, and specific histological subtypes of thyroid cancer. This information helps guide treatment decisions and surgical planning [59].

By combining multiple diagnostic approaches, clinicians can enhance the diagnostic accuracy, risk stratification, and subsequent management decisions for patients with thyroid nodules. Individualized patient evaluation, considering clinical factors, imaging findings, and laboratory parameters, is essential for optimizing patient care and ensuring appropriate treatment plans tailored to each patient’s specific circumstances.

Although the results of our study outline some interesting conclusions, we must also take into consideration the limitations of this research. First, the retrospective design of this study could be considered a limitation. Secondly, although the hospital where the study was conducted serves the western region of Romania, the single-center nature of the study may limit the generalizability of the results. As a future research direction, we proposed a multicenter collaboration for future studies. Thus, due to the mixed population and the much larger number of patients, we could consider the generalization of the results and gain more advanced knowledge regarding the hypotheses discussed.

## 5. Conclusions

In conclusion, several laboratory parameters, including FT3, FT4, ESR, fibrinogen, WBC, and lymphocyte percentage, presented significant differences between the two patient groups. These findings highlight the potential of laboratory parameters in aiding the diagnosis and risk stratification of thyroid nodules. Although routine laboratory tests alone do not provide a definitive diagnosis of thyroid cancer, they play a key role in suggesting and guiding physicians to suspect malignancy in thyroid nodules. This affirmative answer to our question, “Can routine laboratory tests be suggestive in determining suspicions of malignancy in the case of thyroid nodules?” aligns with the results of our study. Integrating these laboratory parameters with other diagnostic modalities, such as imaging and fine-needle aspiration biopsy, is crucial for accurate diagnosis and appropriate management decisions. Future research and advancements in diagnostic techniques will further enhance our ability to accurately diagnose thyroid nodules and optimize patient care.

## Figures and Tables

**Table 1:** Comparison of the patients in the study and control groups in terms of laboratory parameters.

Laboratory Parameters	Study Group (n = 251)	Control Group (n = 594)	*p*-Value *
	Mean ± SD	Mean ± SD	
RBC	4.86 ± 2.73	4.63 ± 0.44	0.189
WBC	7.79 ± 2.15	7.48 ± 1.85	0.0486
neutrophils	5 ± 1.58	4.6 ± 1.53	0.587
lymphocytes	2.09 ± 0.68	2.08 ± 0.69	0.669
monocytes	0.53 ± 0.69	0.5 ± 0.66	0.49
eosinophils	0.14 ± 0.11	0.15 ± 0.16	0.155
basophils	0.035 ± 0.022	0.033 ± 0.027	0.3442
neutrophil%	63.04 ± 8.14	62.17 ± 11.29	0.2092
lymphocyte%	27.07 ± 7.45	28.20 ± 8.41	0.027
PLT	259.2 ± 60.77	261.13 ± 68.85	0.6938
ESR	16.07 ± 6.48	11.04 ± 5.52	0.002
fibrinogen	368.5 ± 97.57	352.9 ± 89.14	0.0238
glycaemia	109.02 ± 27.54	106.30 ± 28.44	0.2043
FT3	5.17 ± 0.7	4.97 ± 1.08	0.0018
FT4	15.5 ± 6.9	14.4 ± 3.45	0.0131
TSH	1.93 ± 1.88	1.98 ± 3.71	0.0581
NLR%	2.64 ± 1.33	2.55 ± 1.44	0.14
NLR	2.64 ± 1.33	2.49 ± 1.94	0.2242

* Independent sample *t*-test; SD = standard deviation; *p*-value < 0.05 = significant.

**Table 2:** Comparison of the laboratory parameters for the main type of cancer in the study group and the control group.

Laboratory Parameters	Study Group	Control Group	*p*-Value *
	Papillary Thyroid Cancer	Nodular Goiter	
	(n = 133)	(n = 394)	
	Mean ± SD	Mean ± SD	
RBC	4.96 ± 3.75	4.62 ± 0.43	0.302
WBC	7.65 ± 2.15	7.54 ± 1.86	0.039
neutrophils	4.92 ± 1.45	4.65 ± 1.53	0.472
lymphocytes	2.03 ± 0.68	2.07 ± 0.69	0.830
monocytes	0.47 ± 0.18	0.53 ± 0.81	0.168
eosinophils	0.14 ± 0.11	0.16 ± 0.16	0.180
basophils	0.03 ± 0.02	0.03 ± 0.03	0.726
neutrophil%	63.80 ± 8.03	62.45 ± 12.24	0.152
lymphocyte%	26.33 ± 7.05	27.94 ± 8.31	0.050
PLT	257.94 ± 59.10	261.87 ± 65.78	0.544
ESR	16.12 ± 6.50	10.95 ± 5.63	0.038
fibrinogen	364.75 ± 90.18	357.46 ± 91.29	0.049
glycaemia	108.46 ± 27.77	107.64 ± 30.89	0.152
FT3	5.15 ± 0.63	5.01 ± 1.00	0.050
FT4	15.85 ± 9.26	14.83 ± 3.14	0.001
TSH	2.10 ± 2.38	2.10 ± 2.38	0.065
NLR%	2.77 ± 1.50	2.55 ± 1.30	0.041
NLR	2.72 ± 1.49	2.48 ± 1.30	0.054

* Independent sample *t*-test; SD = standard deviations; *p*-value < 0.05 = significant.

## Data Availability

The data generated in this study may be requested from the corresponding author.
